# Deletion of the RGD motif in LON-2/glypican is associated with morphological abnormalities

**DOI:** 10.17912/micropub.biology.000376

**Published:** 2021-03-10

**Authors:** Aileen Park, Zhongqiang Qiu, Myeongwoo Lee

**Affiliations:** 1 Baylor University Department of Biology

## Abstract

The *lon-2 *gene in *Caenorhabditis elegans* encodes a heparan sulfate proteoglycan family glypican that negatively regulates the BMP signaling pathway responsible for controlling body length. LON-2 contains multiple functional domains, including an RGD (Arg-Gly-Asp) motif at amino acid number from 348 to 350. A novel mutant allele of *lon-2* was investigated in this study. In this mutant allele,* lon-2(kq348ΔRGD)*, the RGD motif at position 348 was deleted. Another pre-existing mutant allele, *lon-2(e678)*, contains a ~9kb deletion and lacks most of the genomic coding sequence. The *lon-2(e678)* line was used as a reference allele. The novel mutant line was significantly shorter than wild-type animals, suggesting that removal of the RGD motif in LON-2 may improve its ability to inhibit BMP signaling.

**Figure 1.**
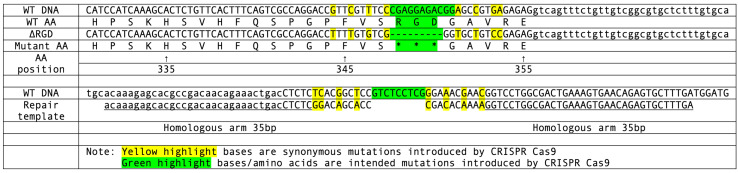
A. Aligned comparison of the wild-type *lon-2* amino acid sequence and the mutant *lon-2(ΔRGD)* sequence containing total deletion of the RGD motif at amino acid position 348. The repair template sequence and synonymous mutation schemes are also included. All sequences are in the 5’ to 3’ direction. *WT: wild-type. *AA: amino acid.

## Description

The *lon-2* gene in *Caenorhabditis elegans* encodes a heparan sulfate proteoglycan family glypican that negatively regulates the BMP signaling pathway responsible for controlling body length (Gumienny **et al.*,* 2007). LON-2 contains multiple functional domains, including an RGD (Arg-Gly-Asp) motif at amino acid number from 348 to 350. RGD motifs are the cell-binding site of the extracellular matrix protein and are known to interact with cell surface receptors of the extracellular matrix, integrins (Margadant and Sonnenberg, 2010). Previous studies show that this RGD motif in LON-2 does not directly regulate BMP, but is essential to the function of soluble LON-2, suggesting that the RGD motif likely utilizes another part of the LON-2 N-terminal core glypican protein functional domain to play a role in BMP signal regulation (Taneja-Bageshwar and Gumienny 2012). These studies indicated that LON-2 acts by binding and sequestering DBL-1 and acting as an RGD-binding protein, modulating the strength of the BMP signal and thereby negatively regulating body size (Gumienny **et al.*,* 2007; Taneja-Bageshwar and Gumienny 2012). Previous analysis demonstrated that *lon-2(e678)* mutants are unable to regulate DBL-1 signaling and as a result of higher doses of DBL-1 are longer than wild-type animals (Gumienny **et al.*,* 2007; Suzuki *et al.*, 1999). A novel mutant allele of *lon-2* was investigated in this study. In this mutant allele, *lon-2(kq348ΔRGD)*, the RGD motif at position 348 was deleted. The previously mentioned pre-existing mutant allele, *lon-2(e678)*, contains a ~9kb deletion and lacks most of the genomic coding sequence (Gumienny *et al.*, 2007). The *lon-2(e678)* line was used as a reference allele in order to identify the presence of morphological abnormalities (the long phenotype) in our novel mutant line. RGD mutant worms as well as wild-type worms were imaged and measured in order to screen for the presence or absence of the long phenotype in the novel mutant line. 45 wild-type animals (n=45) and 27 *lon-2(ΔRGD)* animals (n=27) were imaged and measured in the assay. Wild-type animals had an average body length of 1127.6 µm (SE ± 25.28 µm) and the mutant line had an average body length of 968.6 µm (SE ± 22.98 µm). The 99% confidence intervals for body size of the two lines did not overlap, indicating a statistically significant difference in body length. This suggests that deletion of the RGD motif at position 348 in LON-2 may improve its ability to inhibit BMP signaling, resulting in a shorter body length. The mechanism by which this occurs remains a topic for future investigation, but may happen due to the lack of normal interactions of the RGD motif with integrins and other proteins. Recent research revealing interactions between LON-2 and LIN-17/Frizzled (Saied-Santiago *et al.*., 2017) could guide future research toward investigating the relationship between the LON-2 RGD domain and Frizzled/Wnt signaling.

## Methods

CRISPR target sites were identified using the CRISPR guide RNA Selection Tool (http://genome.sfu.ca/crispr/) in order to ensure that the desired mutations would be created in *lon-2*. The first mutant line *lon-2(ΔRGD)* contains a total deletion of the RGD motif at amino acid position 348 (Figure A). The second mutant line *lon-2(e678)* is a reference allele that has not yet been sequenced, but contains a large ~9kb deletion that removes significant portions of the genomic coding sequence in *lon-2* (Gumienny *et al.*, 2007). The syncytial gonad arms of N2 wild-type worms (P0) were micro-injected with a mixture containing custom template DNA (Temp-4LON2ΔRGD, Figure A), custom crRNA (LON2RGD350), tracrRNA (cat. #1072532), and Alt-R Cas9 nuclease (cat. #1081058) that had been annealed at room temperature (Mello **et al.*,* 1991; Paix *et al.*., 2015). Primers, repair template, sgRNA and Cas9 enzymes were purchased from IDTDNA Inc., Coralville, IA. F1 worms were then isolated and PCR screened using the mutant specific primer (LON2ΔRGD350F) to ensure that the desired mutation had been induced. Both wild-type (LON2RGD350WTF) and mutant specific (LON2ΔRGD350F) primers were then used in PCR screening of the F2 offspring in order to isolate homozygous mutants. The PCR products were then sequenced to confirm that the mutant allele had been successfully created (Psomagen Inc, Rockville, MD). Both mutant lines were backcrossed (2 times) to N2 and the novel RGD mutant line was then studied to characterize phenotype. 45 wild-type and 27 *lon-2(ΔRGD)* animals were imaged and measured to screen for the presence of the long phenotype. Worms were first paralyzed using 10 mM levamisole and were then examined under Nomarski optics using a Nikon NiU epifluorescence microscope (Nikon, Melville, NY). The Nikon Element software was then used to digitally measure the body lengths. Results from this assay were then analyzed using a Mann-Whitney U Test in order to obtain quantitative measures of statistical significance.

**crRNA sequence**

LON2RGD350: ACAGAAACTGACCTCTCTCA

**PCR primers**

LON2RGD350WTF: CCGAGGAGACGGAGCCGTGA

LON2ΔRGD350F: CTTTTGTGTCGGGTGCTGTCC

LON2RGD350SEQF: CCGACCCCTTTCCTCATGATT

LON2RGD350SEQR: TCAAATCCGCCAAATCAGGCT

## Reagents

*lon-2(kq348),* referred to as *lon-2(ΔRGD),* is available upon request.
